# E3 ubiquitin ligase RNF180 prevents excessive PCDH10 methylation to suppress the proliferation and metastasis of gastric cancer cells by promoting ubiquitination of DNMT1

**DOI:** 10.1186/s13148-023-01492-y

**Published:** 2023-05-05

**Authors:** Nannan Zhang, Xiaoliang Gao, Qiangqiang Yuan, Xin Fu, Pengliang Wang, Fenglin Cai, Hui Liu, Jing Zhang, Han Liang, Yongzhan Nie, Jingyu Deng

**Affiliations:** 1 State Key Laboratory of Cancer Biology and National Clinical Research Center for Digestive Diseases, Xijing Hospital of Digestive Diseases, Fourth Military Medical University, Xi’an, 710032 China; 2 Department of Gastric Surgery, Tianjin Medical University Cancer Institute and Hospital, National Clinical Research Center for Cancer, Tianjin’s Clinical Research Center for Cancer and Key Laboratory of Cancer Prevention and Therapy, Tianjin, 300060 China; 3 Guangxi Medical University, Nanning, 530021 Guangxi China; 4 College of Life Sciences, Northwest University, Xi’an, 710069 China

**Keywords:** Gastric cancer, DNA methylation, Tumor suppressor, Ubiquitination, PCDH10, RNF180, DNMT1

## Abstract

**Background:**

Downregulation of certain tumor-suppressor genes (TSGs) by aberrant methylation of CpG islands in the promoter region contributes a great deal to the oncogenesis and progression of several cancers, including gastric cancer (GC). Protocadherin 10 (*PCDH10*) is a newly identified TSG in various cancers and is downregulated in GC; however, the specific mechanisms of *PCDH10* in GC remain elusive. Here, we elucidated a novel epigenetic regulatory signaling pathway involving the E3 ubiquitin ligase RNF180 and DNA methyltransferase 1 (DNMT1), responsible for modulating PCDH10 expression by affecting its promoter methylation.

**Results:**

We revealed that PCDH10 was downregulated in GC cells and tissues, and low PCDH10 expression was correlated with lymph node metastasis and poor prognosis in patients with GC. Additionally, *PCDH10* overexpression suppressed GC cell proliferation and metastasis. Mechanistically, DNMT1-mediated promoter hypermethylation resulted in decreased expression of PCDH10 in GC tissues and cells. Further analysis revealed that RNF180 can bind directly to DNMT1 and was involved in DNMT1 degradation via ubiquitination. Additionally, a positive correlation was found between RNF180 and PCDH10 expression and an inverse association between DNMT1 and PCDH10 expression showed considerable prognostic significance.

**Conclusion:**

Our data showed that RNF180 overexpression upregulated *PCDH10* expression via ubiquitin-dependent degradation of DNMT1, thus suppressing GC cell proliferation, indicating that the RNF180/DNMT1/PCDH10 axis could be a potential therapeutic target for GC treatment.

**Supplementary Information:**

The online version contains supplementary material available at 10.1186/s13148-023-01492-y.

## Background

Gastric cancer (GC) ranks the third-leading cause of cancer-related mortality worldwide, with a dismal prognosis [1]. Studies have shown that the inactivation of tumor suppressor genes (TSGs) caused by promoter hypermethylation contributes to gastric tumorigenesis and cancer progression [2–4]. Therefore, identifying new TSGs that are affected by aberrant DNA hypermethylation and elucidating mechanisms for the epigenetic inactivation of tumor suppressive pathways in gastric cancer may provide potential markers and therapeutic targets for GC treatment.

Protocadherin 10 (PCDH10) has been identified as a functional TSG that is frequently silenced by DNA methylation in various human primary cancers [5–9], and *PCDH10* methylation is an adverse prognostic marker in several cancers [5, 10, 11]. Previous studies revealed that ectopic expression of *PCDH10* suppressed cancer cell malignancy in vitro and restrained tumor growth and metastasis in vivo, substantiating its tumor suppressive roles [5, 12]. However, although the involvement of *PCDH10* has been extensively elucidated in several cancers, the specific mechanisms underlying its silencing via promoter hypermethylation in GC remain elusive.

DNA hypermethylation of promoters at CpG sequences is a well-defined epigenetic hallmark of GC, leading to transcriptional silencing of TSGs and other cancer-related genes [13]. DNA methyltransferase (DNMT) enzymes transfer a methyl group to DNA at the fifth carbon position of cytosine residues, which is a common epigenetic event in the DNA methylation process [2]. There are three main DNMTs: DNMT1, DNMT3A, and DNMT3B, among which DNMT1 is the most abundant and plays a critical role in de novo methylation in the mammalian genome during DNA replication [14, 15]. Recent studies indicated that aberrant expression and function of DNMT1 promoted the progression of GC [16, 17]; however, the specific mechanisms underlying DNMT1 dysregulation in GC are poorly understood.

DNMT1 expression can be regulated at transcriptional and post-transcriptional levels, which influences its catalysis and degradation [18, 19]. Ubiquitination plays a crucial role in posttranslational protein modification and is strongly correlated with several biological and pathological processes in eukaryotes [20]. DNMT1 protein stability is enhanced by inhibiting ubiquitin–proteasome degradation [19, 21–23]. The ubiquitin–proteasome system degrades target proteins tagged with the small protein ubiquitin, via a cascade reaction involving three enzymes: an activating enzyme (E1), an editing enzyme (E2), and a ubiquitin-protein ligase (E3). Recently, it was demonstrated that RING finger proteins, which contain a complex set of E3s containing a RING finger (RNF) domain, are essential for the ubiquitination and degradation of TSGs [24]. RNF180, which shares the RING finger and coiled-coil domains, has been implicated in various biological processes in GC by regulating ubiquitin-dependent degradation of certain proteins [25, 26]. Moreover, RNF180 has been identified as a TSG gene involved in GC lymph-node metastasis and progression [26]. However, whether DNMT1 is subjected to RNF180-mediated ubiquitin–proteasome degradation remains unclear.

Here, we discovered a novel epigenetic regulatory signaling pathway involving the E3 ubiquitin ligase RNF180 and DNMT1 in modulating expression of the TSG PCDH10 by affecting its promoter methylation level using transcriptomic and molecular techniques and in vivo and in vitro experiments. The findings of this study could provide prognostic biomarkers and potential therapeutic targets for effective GC treatment.

## Results

### PCDH10 was significantly downregulated in human GC tissues and significantly correlated with poor prognosis

**Fig. 1:**
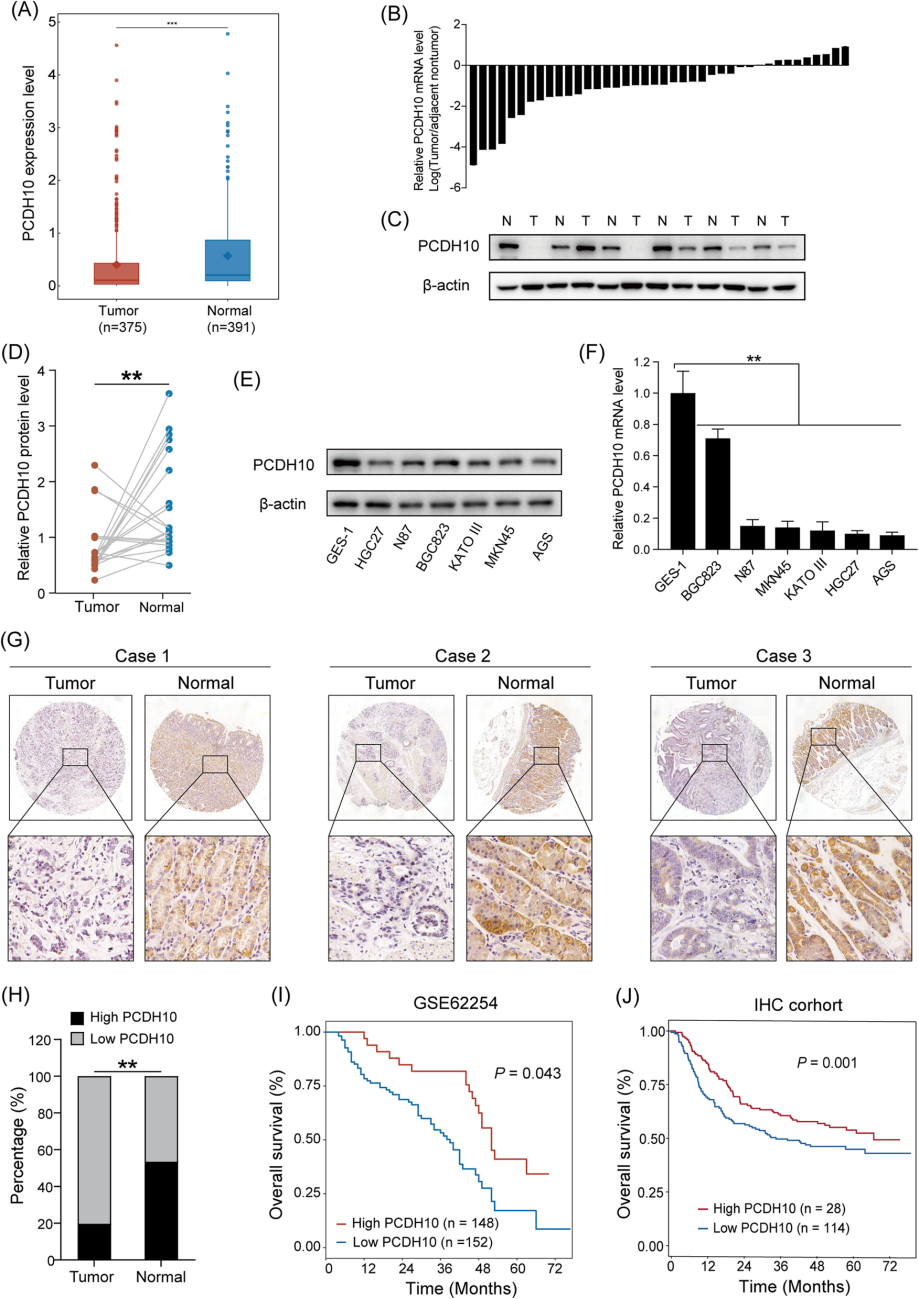
*PCDH10* is downregulated in human gastric cancer (GC) cells, which is associated with poor clinical outcome. **A** *PCDH10* expression in human GC tissue samples from TCGA and Genotype-Tissue Expression (GTEx) database. **B** *PCDH10* mRNA levels in 40 pairs of GC tissues and matched normal tissues. **C**, **D** PCDH10 protein levels in 20 pairs of GC and matched normal tissues. (E, F) PCDH10 mRNA and protein levels in GC cell lines and GES-1. **G**, **H** Representative images of immunohistochemical staining for RNF180 in GC tissues and adjacent nontumor tissues. **I**, **J** Low expression of PCDH10 in human GC tissues is associated with poor clinical outcome. *p < 0.05, ***p < 0.001, ****p < 0.0001. *T* Tumor; *N* Normal

PCDH10 expression in 375 GC and 391 normal samples from The Cancer Genome Atlas (TCGA) and Genotype-Tissue Expression (GTEx) databases was examined. PCDH10 expression was significantly lower (*p* < 0.001) in GC tissues compared with normal gastric tissues (Fig. 1A). Consistent with the microarray data, PCDH10 expression was significantly lower (*p* < 0.001) in GC tissues compared with peritumoral tissues both at the mRNA and protein levels (Fig. 1B–D). Further analysis of GC cell lines confirmed that PCDH10 expression was significantly lower in 6 GC cell lines at both the mRNA and protein levels compared with immortalized GES-1 gastric epithelial cells (Fig. 1E, F, Additional file 1: Fig. S1A). Immunohistochemical analysis was performed to elucidate the correlation between PCDH10 expression and GC progression. Although PCDH10 protein expression was observed mainly in the cytoplasm of GC cells and adjacent nontumor tissues (Fig. 1G), its expression was significantly lower (*χ*^2^ = 34.954, *p* < 0.001) in GC tumor tissues than in adjacent nontumor tissues (Fig. 1G, H). Following the exclusion of samples without survival time and status data from the immunohistochemistry database, 142 patients with GC were divided into two groups based on PCDH10 expression levels. Kaplan–Meier analysis showed that low PCDH10 expression was significantly associated (*p* < 0.01) with poor prognosis (Fig. 1I, J). Moreover, PCDH10 expression levels were identified as a significant independent predictor of prognosis [hazard ratio (HR) = 1.959, *p* = 0.032] with regard to age and pN stage. Detailed results of the univariate and multivariate survival analysis of the 142 GC patients are shown in Table 1. The associations between PCDH10 expression (IHC) and the clinicopathological parameter of GC patients are summarized in Table 2. Notably, PCDH10 expression was negatively associated with the number of lymph node metastasis (*p* = 0.029; pN stage) and tumor size (*p* = 0.043) in GC. Particularly, PCDH10 expression decreased in tumor tissues with increasing number of metastatic lymph node in patients with GC (42.9% for pN0-1 patients, 28.6% for pN2 patients, 21.4% for N3a patients, 7.1% for N3b patients). RNA database analysis further validated the association between PCDH10 expression, clinicopathological parameters, and prognosis in patients with GC (Additional file 2: Table S1). Overall, these results indicate that PCDH10 is downregulated in GC cells and possess prognostic value.

**Table 1:** Univariate and multivariate analyses of factors associated with overall survival

Variable	No. of patients	Univariate analysis		Multivariate analysis	
		5-year OS	*p* value	HR (95% CI)	*p* value
*Gender*					
Male	83	27.7	0.852		
Female	59	25.4			
*Age, years*					
≤ 60	72	33.3	0.016	1.627 (1.042–2.539)	0.032
> 60	70	20.0			
*Tumor location*					
Upper third	35	28.6	0.069		
Middle third	15	28.8			
Lower third	69	40.0			
> 2/3 stomach	23	17.4			
*Tumor size, cm*					
≤ 4	48	41.7	0.002	1.282 (0.818–2.008)	0.278
> 4	94	19.1			
*Lauren classification*					
Intestinal	72	37.5	0.002	0.695 (0.394–1.224)	0.207
Diffuse	70	15.1			
*Type of gastrectomy*					
Distal subtotal	71	28.2	0.403		
Proximal subtotal	18	33.3			
Total	53	22.6			
*pT stage*					
T2	7	42.9	0.009	1.020 (0.615–1.691)	0.939
T3	5	60.0			
T4a	121	25.6			
T4b	9	11.1			
*pN stage*					
N0	15	80.0	< 0.001	1.556 (1.240–1.953)	< 0.001
N1	15	40.0			
N2	28	17.9			
N3a	56	23.2			
N3b	28	7.1			
*PCDH10 expression level (%)*					
Low	113	21.2	0.001	1.959 (1.060–3.620)	0.032
High	29	41.8		1 (Ref)	

*CI* Confidence interval; *HR* hazard ratio; *No.* Number; *OS* Overall survival; *Ref* Reference

Variables with *p* values less than 0.1 were included in the multivariate analysis

**Table 2:** Correlations between PCDH10 and clinicopathological features in 142 gastric cancer patients in IHC database

Variable	Number of patients	Chi-square value		*p* value*
	PCDH10^Low^	PCDH10^High^		
*Gender*				
Male	64	19	0.489	0.484
Female	50	9		
*Age, years*				
≤ 60	59	13	1.819	0.177
> 60	55	15		
*Tumor location*				
Upper third	28	7	1.866	0.610
Middle third	11	4		
Lower third	57	12		
> 2/3 stomach	18	5		
*Tumor size, cm*				
≤ 4	33	14	4.089	0.043
> 4	81	14		
*Lauren classification*				
intestinal	53	19	1.398	0.237
diffuse	61	9		
*Type of gastrectomy*				
Distal subtotal	57	12	0.146	0.930
Proximal subtotal	14	4		
Total	43	12		
*pT stage*				
T2	5	2	1.605	0.828
T3	3	2		
T4a	97	24		
T4b	9	0		
*pN stage*				
N0	10	5	10.237	0.029*
N1	10	5		
N2	19	9		
N3a	50	6		
N3b	25	3		

A chi-square test was used for comparing groups between low and high PCDH10 expression

*p < 0.05 was considered significant

### Decreased expression of PCDH10 in GC cells and tissues was associated with promoter hypermethylation

**Fig. 2:**
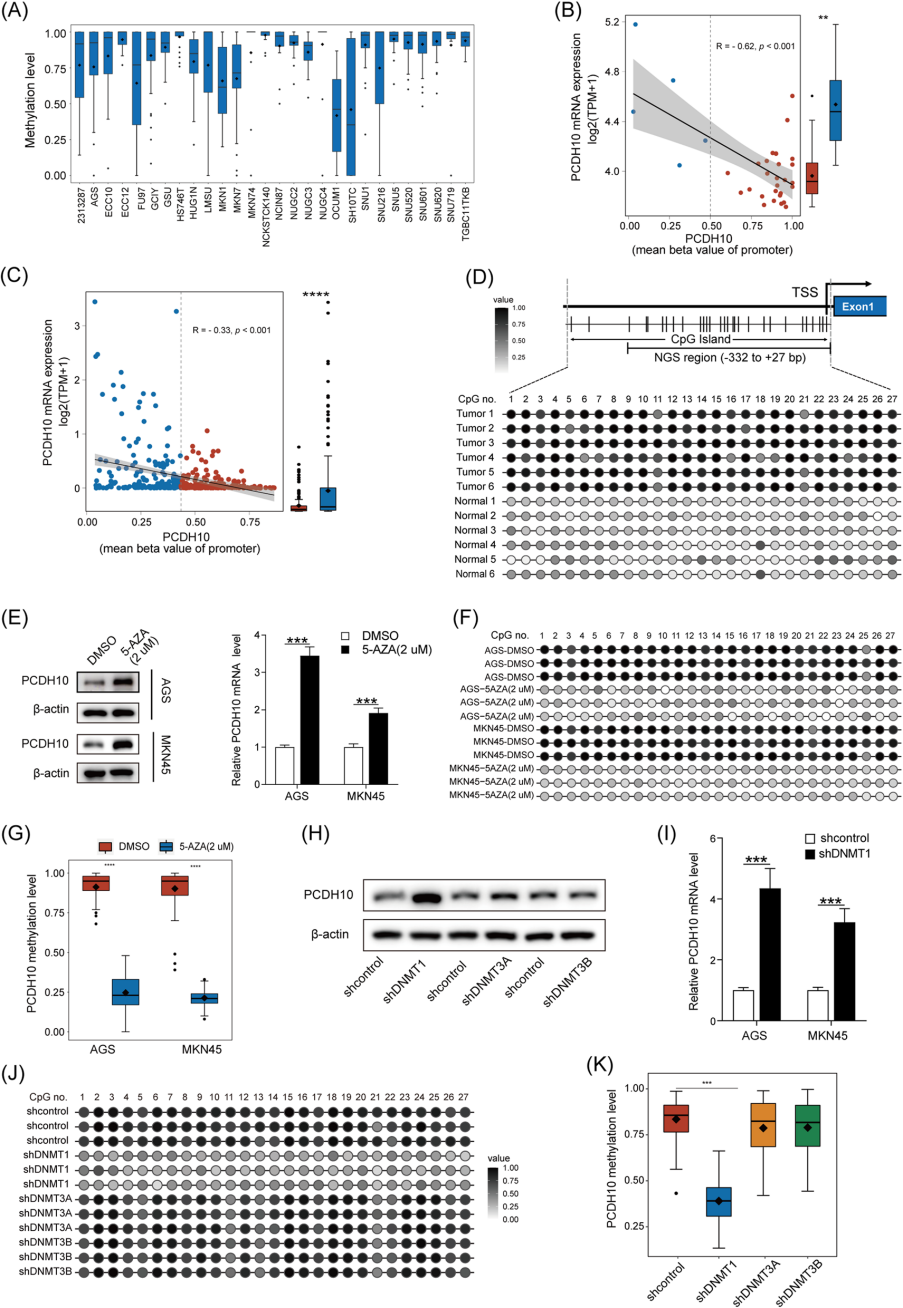
Decreased expression of PCDH10 is attributed to DNMT1-mediated promoter hypermethylation. **A** The global methylation level of PCDH10 in gastric cancer (GC) cells from the Broad Institute CCLE databases. **B** High *PCDH10* methylation levels were associated with low mRNA levels from the Broad Institute CCLE database. **C** *PCDH10* methylation level in GC tissues and adjacent nontumor tissues was confirmed by NGS methylation analysis. **D** High *PCDH10* methylation levels were associated with low mRNA levels from the TCGA database. **E**, **F**, **G** Effect of 5-AZA treatment on mRNA, protein expression and methylation level of PCDH10. **H**, **I** Western blot and qPCR confirmed that DNMT1 knockdown increased the protein and mRNA expression of *PCDH10*. **J**, **K** NGS methylation analysis showed that DNMT1 knockdown suppressed the global methylation level of PCDH10 in AGS cells. **p < 0.01, ***p < 0.001

To determine the role of promoter methylation in silencing of PCDH10, the expression and methylation status of PCDH10 in human GC tissues were investigated using the epigenome-wide association studies (EWAS) database and Broad Institute CCLE databases. Compared with adjacent normal tissues, PCDH10 methylation was significantly higher in GC tissues and cells (Fig. 2A and Additional file 1: Fig.S1B, C). Moreover, high PCDH10 methylation levels were associated with low mRNA expression levels in both GC tissues and cells (Fig. 2B, 2C). NGS methylation analysis was performed to determine PCDH10 methylation status. The CpG islands and selected region for NGS methylation analysis in the PCDH10 promoter region were − 332 to + 27 bp relative to the transcription start site, containing 27 CpG dinucleotides. PCDH10 displayed full or partial promoter methylation in tumor tissues, whereas little or no methylation was observed in the adjacent nontumor tissues (Fig. 2D and Additional file 1: Fig. S1D). Furthermore, treatment with DNMT inhibitor 5-aza-2′-deoxycytidine (2 uM) resulted in the decrease of PCDH10 DNA methylation and restored PCDH10 mRNA and protein expression in the examined GC cell lines (Fig. 2E–G), further supporting for the conclusion that PCDH10 silencing is regulated by promoter methylation in GC cell lines.

### PCDH10 expression was mainly silenced by DNMT1

DNMT1, DNMT3A, and DNMT3B knockdown AGS cells were generated to assess the role of DNMTs in mediating PCDH10 promoter methylation. Downregulation of DMMT1 but not DNMT3A and DNMT3B significantly restored PCDH10 expression (Fig. 2H, I, and Additional file 1: Fig. S1E, F), which was further confirmed by NGS methylation analysis (Fig. 2J, K). Overall, these results indicated that DNMT1 was involved in silencing PCDH10 expression in GC cells.

### PCDH10 inhibited GC cell proliferation in vitro and in vivo

**Fig. 3:**
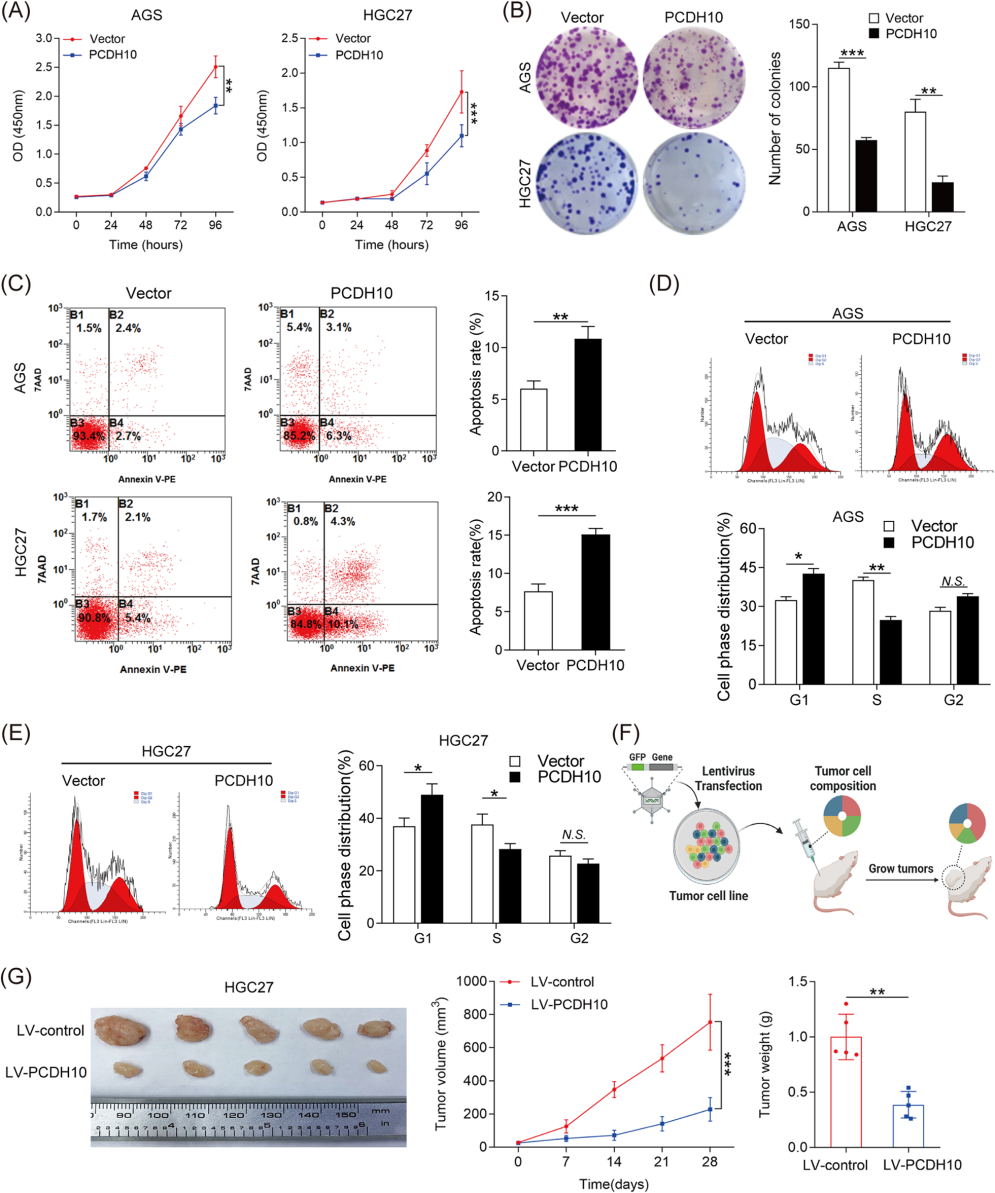
PCDH10 overexpression inhibits GC progression in vitro and in vivo. **A**, **B** CCK-8 assay and colony formation was performed to determine the effect of *PCDH10* overexpression on cell proliferation. **C**, **D**, **E** The effects of *PCDH10* overexpression on cellular apoptosis and cell cycle were detected by flow cytometry. **F**, **G** PCDH10 stably overexpressed HGC27 cells and control cells were injected subcutaneously into nude mice (n = 5). *p < 0.05, ***p < 0.001, ****p < 0.0001, *N.S.*, not significant

To elucidate the biological roles of PCDH10 in GC cell proliferation, AGS and HGC27 cells overexpressing PCDH10 were generated. CCK-8 and colony formation assays demonstrated that PCDH10 overexpression significantly inhibited GC cell growth and colony formation in AGS and HGC27 cells (Fig. 3A, B). Additionally, upregulation of PCDH10 enhanced apoptosis and significantly retarded cell cycle progression in AGS and HGC27 cells (Fig. 3C, D, E). Moreover, apoptosis markers PARP, cleaved PARP, caspase 3 and cleaved caspase 3 expression levels were detected by western blotting experiments. The results demonstrated that PCDH10 overexpression led to an elevated expression of the cleaved form of caspase-3 and poly (ADP-ribose) 2 polymerase (PARP) in HGC27 and AGS cells treated with staurosporine, which is an apoptosis-inducing reagent (Additional file 1: Fig. S2A). Furthermore, HGC27 cells stably transduced with lentiviral PCDH10 (LV-PCDH10) or negative control (LV-control) were transplanted into nude mice to determine whether PCDH10 regulates GC cell proliferation in vivo. The LV-PCDH10 group had significantly lower tumor volumes and weights compared with the negative control group (Fig. 3F, G). Collectively, these results indicated that PCDH10 suppressed GC cell proliferation both in vitro and in vivo.

### PCDH10 inhibited GC cell migration and invasion in vitro and metastasis in vivo

**Fig. 4:**
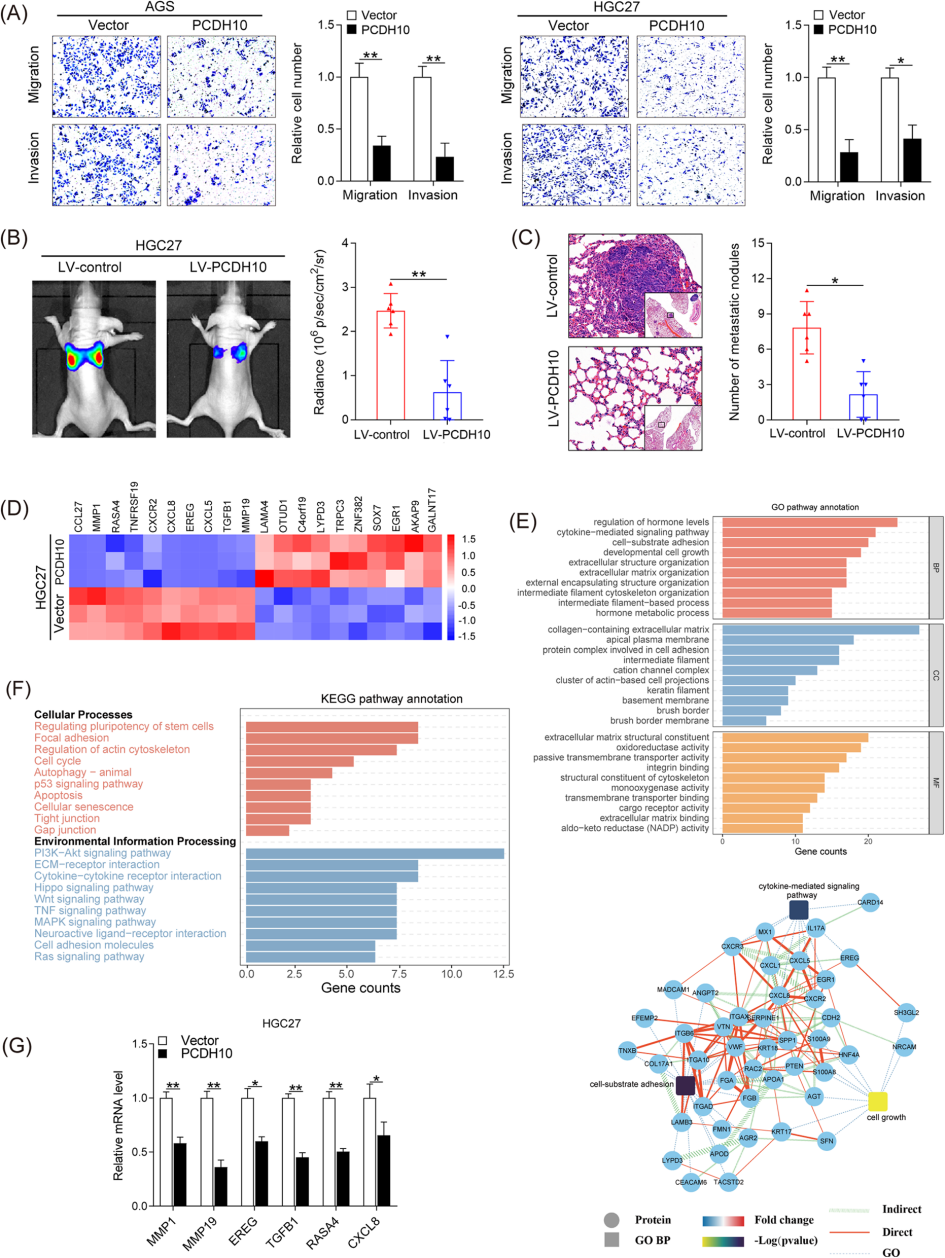
PCDH10 overexpression inhibits the migratory and invasive abilities of GC cells in vitro and in vivo. **A** Transwell assay was performed to determine the effects of *PCDH10* overexpression on cellular invasion and migration. **B** Representative bioluminescence images of the different groups are shown at 8 weeks after tail vein injection (left). The radiance was collected and calculated (right). **C** Representative hematoxylin and eosin (H&E) staining images of lung tissue sections from different groups (left). The number of lung metastatic foci was calculated (right). Scale bars: 200 μm (main) and 50 μm (insert). **D** Heatmap showing differentially expressed genes in *PCDH10*-overexpressing HGC27 cells. **E** The interaction diagram of proteins involved cell growth, cytokine-mediated signaling pathway, and cell-substrate adhesion. **F** The significantly dysregulated pathways among PCDH10 overexpression and control groups in different KEGG categories. **G** RT-PCR analysis of target genes in indicated cells. *p < 0.05, ***p < 0.001, ****p < 0.0001, *N.S.* Not significant

Since elevated PCDH10 levels correlated with lymph node metastasis (Table 2), we speculated that PCDH10 may be involved in GC cell metastasis. The transwell migration and invasion assays revealed that PCDH10 overexpression significantly suppressed the migration and invasive ability of AGS and HGC27 cells (Fig. 4A). To validate the in vitro findings, LV-PCDH10-transfected HGC27 cells were injected in the tail vein of nude mice. Mice injected with LV-PCDH10-transfected HGC27 cells mice exhibited reduced bioluminescence intensity in the lung and decreased lung metastases (Fig. 4B, C). These results confirmed that PCDH10 significantly suppressed GC cell migration and invasion in vitro and metastasis in vivo.

Furthermore, RNA sequencing of PCDH10-overexpressing HGC27 cells and control cells were performed to elucidate the mechanisms by which PCDH10 inhibits GC progression (Fig. 4D and Additional file 1: Fig.S2B, C). Gene ontology enrichment analysis showed that most differentially expressed genes between the two groups (PCDH10 overexpression and control groups) were involved in the cell growth, cytokine-mediated signaling pathway, and cell-substrate adhesion (Fig. 4E). Also, KEGG pathway analysis of the DEGs highlighted focal adhesion, apoptosis and cell cycle processes and cytokine − cytokine receptor interaction pathway (Fig. 4F). Moreover, genes involved in cell proliferation [epiregulin (EREG)), invasion (matrix metalloproteinase (MMP) 1 and 19], angiogenesis and lymph angiogenesis [transforming growth factor beta 1 (TGFB1) and RAS p21 protein activator 4 (RASA4)], and chemotaxis [C-X-C motif chemokine ligand 8 (CXCL8)] were significantly downregulated in PCDH10-overexpressing GC cells (Fig. 4G), indicating that PCDH10 may suppress GC progression by downregulating the expression of several critical oncogenes.

### RNF180 restored PCDH10 expression by inhibiting DNMT1

**Fig. 5:**
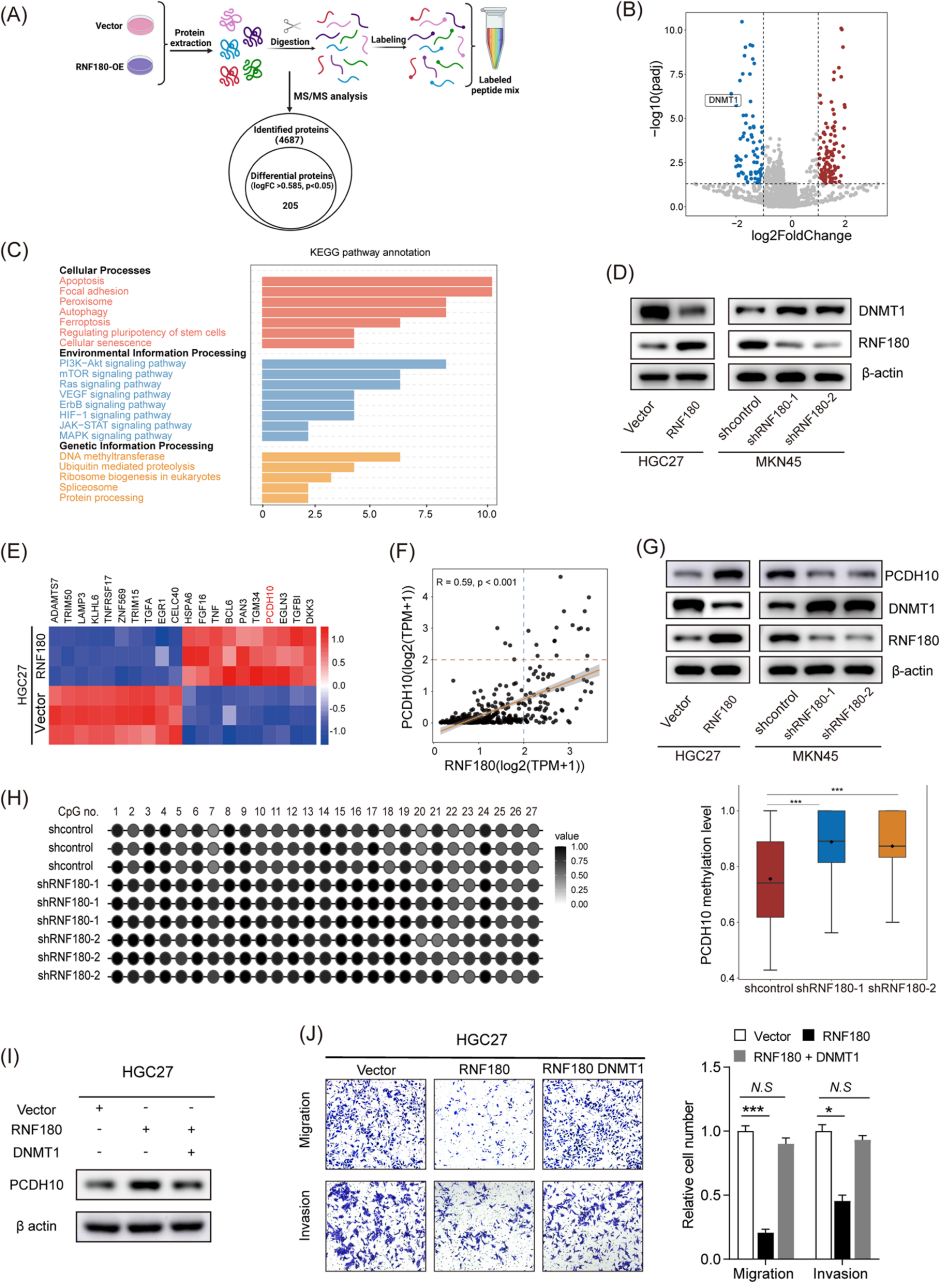
RNF180 restored PCDH10 expression by inhibiting DNMT1. **A**, **B** Volcano plot of altered gene expression patterns in RNF180 overexpressed HGC27 cells identified from iTRAQ quantitative analysis. **C** The significantly dysregulated pathways among RNF180 overexpression and control groups in different KEGG categories. **D** Western blot showed downregulation or upregulation of RNF180 expression changed the DNMT1 expression in GC cells. **E** Heatmap of 20 dysregulated genes identified in HGC27-RNF180 and control cells using gene chip detection analysis. (F) Spearman’s correlation analysis of TCGA database indicated RNF180 expression was positively correlated with PCDH10 expression. **G** Western blot showed downregulation or upregulation of RNF180 expression changed the PCDH10 expression in GC cells. **H** PCDH10 methylation increased after RNF180 knockdown, as indicated by NGS methylation analysis. **I** Western blot analysis confirmed that DNMT1 overexpression rescued RNF180 overexpression-induced increase in PCDH10 expression. **J** Transwell assays revealed that DNMT1 upregulation partially countervailed the inhibitive effect of RNF180 overexpression on the invasion and migration of HGC27 cells. **p < 0.01, ***p < 0.001

Several studies have been performed to identify biomarkers for GC in the last decade [5, 26, 27], and RNF180 has been shown to act as a TSG in GC [26]. Moreover, iTRAQ quantitative proteomics analysis showed that DNMT1 was associated with RNF180 (Fig. 5A, B and Additional file 1: Fig.S3A, B). Gene ontology enrichment analysis and KEGG pathway analysis also revealed that differentially expressed genes between the two groups (RNF180 overexpression and control groups) were involved in the ubiquitin binding, methyltransferase activity and genetic information processing of DNA methyltransferase and ubiquitin mediated proteolysis (Fig. 5C and Additional file 1: Fig. S3C). Further analysis indicated that RNF180 overexpression markedly reduced DNMT1 protein expression, whereas RNF180 knockdown enhanced DNMT1 expression; however, neither RNF180 overexpression or inhibition influenced DNMT1 mRNA levels (Fig. 5D and Additional file 1: Fig. S3D). These results indicated that RNF180 regulated DNMT1 expression at the post-transcriptional level.

Additionally, HGC27 cells transfected with RNF180-overexpression plasmid had significantly higher PCDH10 expression than those transfected with the control vector using an Affymetrix GeneChip arrays (Fig. 5E). Moreover, RNF180 expression was significantly correlated (*r* = 0.59, *p* < 0.001) with PCDH10 expression, based on correlation analysis of data obtained from TCGA database (Fig. 5F). Since PCDH10 is mainly regulated by DNMT1, we hypothesized that RNF180 could regulate PCDH10 expression via DNMT1. Consistent with our hypothesis, western blotting analysis and qRT-PCR analysis showed that RNF180 overexpression increased PCDH10 expression (Fig. 5G and Additional file 1: Fig.S3E). Additionally, NGS assay showed that RNF180 knockdown increased PCDH10 methylation levels in MKN45 cells compared with the control group (Fig. 5H). As RNF180 is a RING-domain E3 ubiquitin ligase, and the RING domain is reported to be responsible for this kind of E3 ligase activity. Based on this, we constructed a RING-domain truncated mutants of RNF180 and the corresponding western blotting experiments were performed. The results revealed that mutation in the RING domain of RNF180 did not affect the protein level of DNMT1 and PCDH10 (Additional file 1: Fig.S3F). Furthermore, a rescue assay was performed to verify whether RNF180 regulated PCDH10 expression via DNMT1. Expectedly, DNMT1 expression significantly suppressed RNF180-induced increase in PCDH10 expression in HGC27 cells (Fig. 5I). Transwell, CCK-8, and colony formation assays revealed that DNMT1 upregulation partially reversed RNF180 overexpression-induced inhibition of HGC27 cell proliferation, migration, and invasive ability (Fig. 5J and Additional file 1: Fig. S3G, H). Overall, these results indicated that RNF180 restored PCDH10 expression by inhibiting DNMT1.

### RNF180 enhanced ubiquitination and degradation of DNMT1

**Fig. 6:**
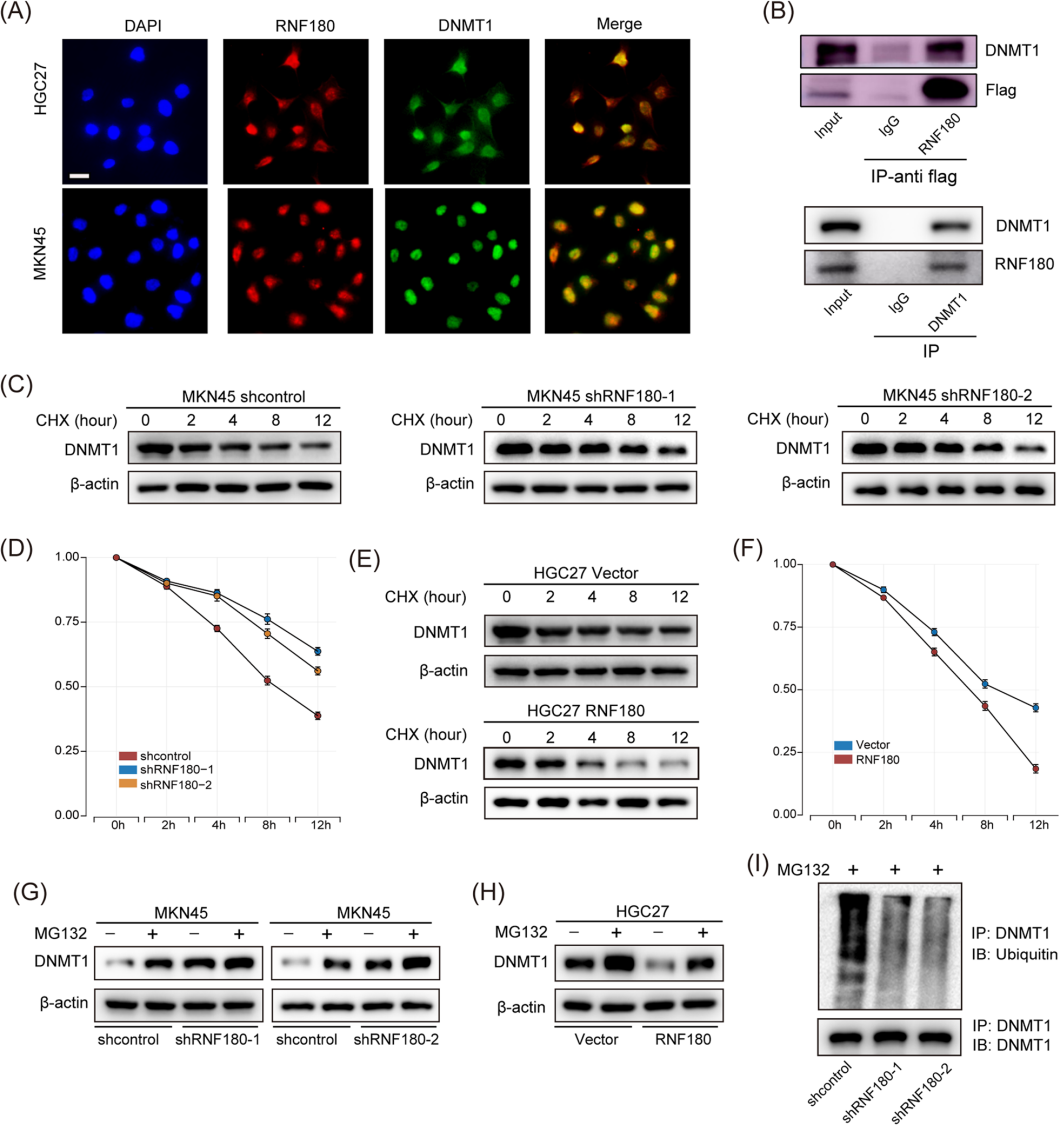
RNF180 enhances DNMT1 ubiquitination and degradation. **A** Immunofluorescence assay showed RNF180 and DNMT1 were co-localized in MKN45 and HGC27 cells. Scale bar: 20 μm. **B** Coimmunoprecipitation (Co-IP) assay was performed to detect the interaction between RNF180 and DNMT1. **C**, **D** DNMT1 degradation was suppressed in MKN45-shRNF180 cells compared with that in MKN45-shcontrol cells, as indicated by CHX chase assay. **E**, **F** DNMT1 degradation was higher in HGC27-RNF180 cells compared with that in HGC27-vector cells, as indicated by CHX chase assay. **G** DNMT1 expression was detected by western blot in MKN45-shcontrol and MKN45-shRNF180 cells after being treated with DMSO or MG132. **H** DNMT1 expression was detected by western blot in HGC27-vector and HGC27-RNF180 cells after being treated with DMSO or MG132. **I** Western blot images showing DNMT1-associated ubiquitination (Ubi) in control and RNF180-knockdown MKN45 cells treated with MG132

RNF180 is an E3 ubiquitin ligase which functions by increasing ubiquitin–proteasome degradation of targeted proteins. Therefore, we hypothesized that RNF180 can alter DNMT1 stability through ubiquitination, resulting in its degradation through the proteasome pathway. To confirm this hypothesis, immunofluorescence, coimmunoprecipitation (Co-IP), and cycloheximide chase assays were performed to determine the interaction between RNF180 and DNMT1. Immunofluorescence assay showed that RNF180 and DNMT1 were co-localized in MKN45 and HGC27 cells (Fig. 6A). Moreover, Co-IP assay verified that RNF180 interacted directly with DNMT1 (Fig. 6B). Furthermore, cycloheximide chase assay showed that RNF180 depletion was associated with an increase in the half-life of DNMT1 (Fig. 6C, D), confirming that DNMT1 is a substrate of RNF180. Similarly, DNMT1 half-life decreased in HGC27 cells transduced with RNF180-overexpressing lentiviruses (Fig. 6E, F), whereas treatment with the proteasomal inhibitor MG132 significantly increased DNMT1 expression, with RNF180 downregulation producing the same effect in MKN45 cells (Fig. 6G). However, RNF180 overexpression partly reversed MG132-induced increase in DNMT1 expression in HGC27 cells (Fig. 6H). Additionally, RNF180 lacking the RING domain failed to reverse MG132-induced increase in DNMT1 expression in HGC27 cells (Additional file 1: Fig. S3I). Moreover, Co-IP assay indicated that DNMT1 ubiquitination was suppressed by RNF180 knockdown in MKN45 cells (Fig. 6I). Overall, these results suggest that RNF180 regulated DNMT1 stability via proteasome-mediated mechanisms.

### PCDH10 expression was positively correlated with RNF180 expression and negatively correlated with DNMT1 expression in human GC tissues

**Fig. 7:**
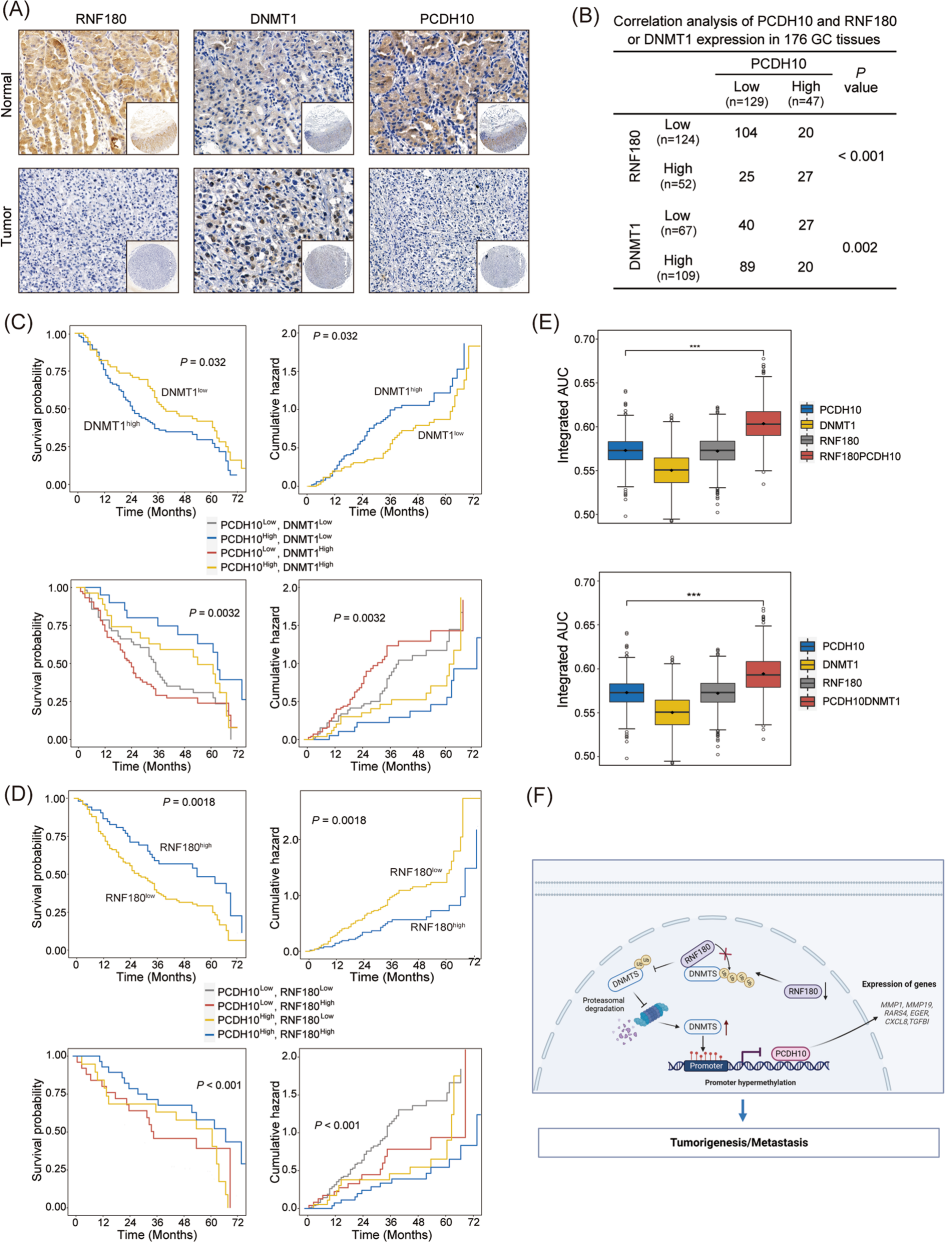
*PCDH10* expression is positively correlated with RNF180 and negatively associated with DNMT1 expression in human gastric cancer (GC) tissues. **A** Representative immunohistochemical images of PCDH10, RNF180, and DNMT1 in GC tissues and adjacent non-tumorous tissues. Scale bars: 200 μm (main) and 20 μm (insert). **B** The correlation between PCDH10 expression and RNF180 or DNMT1 expression in GC tissues. **C** Kaplan–Meier survival curves for overall postoperative survival and cumulative death hazard showed that GC patients with high-expression of DNMT1 and low expression of PCDH10 showed poorer prognosis. **D** Kaplan–Meier survival curves for overall postoperative survival and cumulative death hazard showed that GC patients with low-expression of both DNMT1 and PCDH10 showed poorer prognosis. **E** The predictive accuracy for overall survival based on the iAUC with 1000 × bootstrap resampling for the five parameters is shown as a box plot. The iAUC indicates integrated area under the ROC curve. **F** Model depicting the mechanism through which RNF180 regulate PCDH10 expression via the ubiquitin-dependent degradation of DNMT1, this model is created with https://www.biorender.com/

Immunohistochemical analysis showed that PCDH10 expression was positively correlated with RNF180 expression but negatively correlated with DNMT1 expression (Fig. 7A, B). DNMT1 overexpression and low expression of RNF180 were associated with poor prognosis (Fig. 7C, D) and aggressive tumor behavior (Additional file 2: Tables S2, 3). Patients were divided into four groups based on PCDH10 and RNF180, and PCDH10 and DNMT1 expression levels, respectively. Kaplan–Meier analysis showed that patients with co-expression of PCDH10 and RNF180 had the lowest death rates and longest overall survival times (Fig. 7C, D). Moreover, survival analysis showed that patients with low expression of RNF180 and high expression of DNMT1 had the worst prognosis (Fig. 7C, D). Regarding predictive accuracy, co-expression of PCDH10 and RNF180 or co-expression of PCDH10 and DNMT1 had superior predictive accuracy for overall survival of GC patients than single expression of RNF180, PCDH10, or DNMT1 (Fig. 7E).

## Discussion

Epigenetic changes contribute to cancer progression by hypermethylation and silencing of target TSGs [28], and PCDH10 hypermethylation has been shown to cause PCDH10 downregulation in certain cancers [5]. In the present study, PCDH10 was downregulated in GC samples, which was attributed to promoter hypermethylation. Additionally, PCDH10 methylation was regulated by DNMT1; however, RNF180 treatment promoted PCDH10 expression by mediating ubiquitin-dependent proteasome degradation of DNMT1. Particularly, PCDH10 expression inhibited the proliferation and metastasis of GC cells in vitro and in vivo by suppressing the expression of several oncogenes. The regulatory relationships between RNF180/DNMT1 and PCDH10 were further confirmed in clinical samples and were associated with prognosis in patients with GC.

Accumulating evidence has shown that PCDH10 is a TSG in several cancers, and its ectopic expression can inhibit tumorigenesis, suppress cell metastasis, and induce apoptosis [5–11]. Several molecular mechanisms have been proposed for the tumor suppressive role of PCDH10. For example, PCDH10 was directly engaged in the negative regulation of the epidermal growth factor receptor signaling pathway [9], resulting in tumor suppression. Additionally, PCDH10 suppressed MM cell proliferation by negatively regulating the Wnt/β-catenin/Bcl-9 signaling pathway [29], and a new PCDH10-Wnt/-catenin-MALAT1 regulatory axis was shown to contribute to the progression of endometrioid endometrial cancer [12]. In the present study, PCDH10 overexpression significantly decreased the expression of genes associated with tumor progression and metastasis, including EREG, MMP1, MMP9, TGFB1, RASA4, and CXCL8, in GC cells [30–34]. Blood-based liquid biopsy to detect and monitor cancer might lead to early diagnosis of cancer and improve clinical oncological decision-making [4]. Currently, plasma RNF180 and PCDH10 methylation levels have been used as biomarkers for the diagnosis and screening for GC and CRC, respectively [10, 35]. Regarding the clinical significance of PCDH10, low PCDH10 levels have been observed in various cancers and have been considered a promising prognostic marker in patients with colorectal cancer [11], bladder cancer [36], pancreatic ductal adenocarcinoma [37], breast cancer [38], and diffuse large B-cell lymphoma [39]. In the present study, PCDH10 expression was significantly downregulated in GC tissues compared with matched peritumoral tissues, and low PCDH10 protein level was correlated with increased lymphatic metastasis and poor prognosis in patients with GC. These results indicate that PCDH10 is a good prognostic predictor of metastasis and malignancy in GC.

DNA methylation is a reversible enzyme-mediated modification that contributes to cancer progression. DNMT1, the major DNA methyltransferase, is responsible for maintaining DNA methylation and is recognized as the ‘maintenance methyltransferase’ [40]. Previous studies have shown that high DNMT1 expression is inversely related to poor survival outcome in patients with GC and other types of cancers [40–43]. Moreover, DNMT1 expression in gastric carcinomas and solid tumors has been shown to be significantly associated with chemotherapy response [17]. DNA demethylating agents have shown promising therapeutic effects in hematological malignancies and several solid tumors [44]. In the present study, DNMT1 was significantly upregulated in the GC samples, and negatively association between DNMT1 and PCDH10 expression. Additionally, DNMT1 knockdown but not DNMT3A and DNMT3B knockdown restored PCDH10 expression and reduced PCDH10 methylation level. Moreover, DNMT1 expression was negatively correlated with PCDH10 expression in human GC tissues, and PCDH10 and DNMT1 co-expression was an independent prognostic factor for overall survival in patients with GC. These results suggest that DNMT1-mediated hypermethylation of PCDH10 promoter may contribute to PCDH10 downregulation in GC.

Regarding the mechanism underlying the overexpression of DNMT1 in GC, this study identified a post-translational modification pathway mediated by RNF180. The ubiquitin–proteasome system, one of the most important regulators of protein metabolism, plays an important role in tumor initiation and development [24]. E3 ubiquitin ligases are crucial for ubiquitin proteasomal degradation by determining the particularity and timing of ubiquitination as well as subsequent degradation of substrates [24, 45]. Previous studies showed that DNMT1 expression was regulated by adjusting its ubiquitination and acetylation level [21, 46–48]. As an E3, RNF180 interacts physically with multiple target proteins to achieve ubiquitination and proteasomal degradation. Moreover, RNF180 has previously been shown to act as a suppressor gene to inhibit GC progression. In the present study, RNF180 was associated with decreased ubiquitination and degradation of DNMT1, indicating that RNF180 regulated DNMT1 protein stability by modulating its ubiquitination levels. However, no specific ubiquitination sites have been identified, indicating the need for further studies.

In the present study, RNF180 significantly decreased PCDH10 methylation level and increased PCDH10 expression. A positive correlation was observed between RNF180 and PCDH10 expression levels, and their positive co-expression was associated with favorable clinical prognosis in patients with GC. Based on experimental and clinical evidence, we speculated that RNF180 might affect GC properties by controlling DNMT1/PCDH10 signaling (Fig. 7F). Consistent with our hypothesis, it was observed that RNF180 indirectly regulated PCDH10 expression in GC via DNA methyltransferase, providing a better understanding of the epigenetic mechanisms of GC.

## Conclusions

The results of this study showed that the E3 ubiquitin ligase RNF180 regulated PCDH10 expression by affecting its promoter methylation through the activity of DNMT1. Additionally, PCDH10 was downregulated in GC, which was associated with a poor prognosis in patients with GC. Overall, the present study uncovers a new mechanism by which PCDH10 DNA methylation contributes to GC progression and initiation, indicating that GC treatment could be improved by targeting this protein with DNA demethylating agents. Moreover, these results suggest that PCDH10 and RNF180 could be potential biomarkers for GC diagnosis.

## Methods

### Patients and tissues

A total of 142 paraffin-embedded advanced gastric adenocarcinoma (pT2–4NanyM0) specimens and paired adjacent noncancerous tissue samples (> 5 cm from cancer tissue) and their associated clinical data were collected from GC patients who underwent radical gastrectomy between September 2011 and July 2014 to evaluate the prognostic value of PCDH10. Additionally, 176 paraffin-embedded advanced gastric adenocarcinoma (pT2–4NanyM0) and matched adjacent noncancerous tissues (> 5 cm from cancer tissue) were collected between August 2004 and December 2007 to construct tissue microarray (TMA) to examine the correlation between RNF180 and PCDH10 expression. Furthermore, another 40 pairs of frozen GC and adjacent normal mucosa samples collected from GC patients from September 2019 to July 2020 were used for real-time (RT)-PCR and western blot analysis. The inclusion and exclusion criteria are provided in the Supplemental Material. All the enrolled patients were restaged according to the 8th edition of the TNM classification for GC. This study was approved by the Ethics Committee of Tianjin Medical University Cancer Institute & Hospital and was conducted according to the Declaration of Helsinki.

### Generation of stable cell lines

Lentiviral vector containing RNF180, DNMT1, DNMT3A, and DNMT3B short hairpin RNAs (shRNAs) were transfected into gastric cancer cells to construct the knockdown cell lines. Additionally, lentiviral vector containing PCDH10 and RNF180 DNA sequence (LV-PCDH10 and LV-RNF180) was transfected into gastric cancer cells to construct RNF180 and PCDH10 overexpression cell line. The lentiviral vectors and negative control were constructed by Shanghai GeneChem (Shanghai, China). Target cells (1 × 10^5^) were infected with lentivirus transducing units followed by puromycin treatment for 2 weeks. The shRNA sequences were as follows: RNF180 shRNA-1, CCAGAATGGATAAGCTGCCTA; shRNA-2, ATGGAGTATCTTGAGAATCAA; and shRNA-3, TAGTCAAGAGGAAACAAGTAT; and DNMT1, GCCCAATGAGACTGACATCAA; DNMT3A, CCGGCTCTTCTTTGAGTTCTA; and DNMT3B, AGATGACGGATGCCTAGAG. (Additional file 1: Fig. S4).

### Ubiquitination assay

MKN45-shcontroland MKN45-shRNF180 stable cells were incubated with 10 μM MG132 (HY-13259, MCE) for 12 h, lysed, and subjected to immunoprecipitation with anti-DNMT1 antibodies (GTX116011; GeneTex). Ubiquitination of substrates was analyzed by SDS-PAGE and western blotting using an anti-ubiquitin antibody (Ab134953; Abcam).

### Statistical analysis

Statistical analyses were performed using SPSS (version 22.0), GraphPad Prism (version 8.0 software), and R software (version 4.2.1). The differences between two or more groups were analyzed using Student’s *t*-test and one-way ANOVA, unless otherwise stated. Pearson’s or Spearman’s correlation analysis was performed to determine the relationship between variables. Kaplan–Meier log-rank test (Mantel-Cox) was utilized to compare survival data, and Cox proportional hazards regression models were used to conduct univariate and multivariate analyses. Mean values were considered statistically significant at *p* < 0.05. Additional details of the methods are described in Additional file 3.

## Supplementary Information

**Additional file 1: Fig. S1.** **A** PCDH10 protein levels in GC cell lines and GES-1. **B** The global methylation level of PCDH10 in gastric cancer cells from the Broad Institute CCLE databases. **C** The global methylation level of PCDH10 in gastric cancer tissues, adjacent non-tumor tissues from EWAS database. **D** The global methylation status of PCDH10 in gastric cancer tissues, adjacent non-tumor tissues confirmed by the NGS methylation analysis. **E, F** Western blot and qPCR confirmed that DNMT1 knockdown increased the protein and mRNA expression of PCDH10. **Fig. S2. A** PCDH10 overexpression led to an elevated expression of the cleaved form of caspase- 3 and poly (ADP-ribose) 2 polymerase (PARP) in HGC27 and AGS cells treated with staurosporine, which is an apoptosis-inducing reagent. **B** PCA for the expression profiles to distinguish the two groups (PCDH10 overexpression and control groups). **C** Volcano plot of altered gene expression patterns in PCDH10 overexpressed HGC27 cells identified from RNA-seq analysis. **Fig. S3. A** PCA for the expression profiles to distinguish the two groups (RNF180 overexpression and control groups. **B** Heatmap of 30 dysregulated genes identified from iTRAQ quantitative analysis. **C** The significant GO terms in the enrichment analysis of 30 dysregulated genes identified from iTRAQ quantitative analysis. **D** qPCRanalyses were used to detect the mRNA expression of DNMT1 in RNF180 overexpressed HGC27 cells and RNF180 silencing MKN45 cells. **E** qPCR analyses were used to detect the mRNA expression of PCDH10 in RNF180 overexpressed HGC27 cells. **F** Mutation in the RING domain of RNF180 did not affect the protein level of DNMT1 and PCDH10. **G, H** CCK8 and colonyforming assays confirmed that DNMT1 upregulation partially countervailed the inhibitive effect of RNF180 overexpression on the proliferation of HGC27 cells. **I** RNF180 lacking the RING domain failed to reverse MG132-induced increase in DNMT1 expression in HGC27 cells. **Fig. S4. A** Stable PCDH10 overexpressed HGC27 and AGS cells were established. **B, C** Stable RNF180 overexpressed HGC27 cells (**B**) and stable RNF180 knockdown MKN45 cells (**C**) were established. **D, E, F** Stable DNMT1 knockdown AGS cells (**D**), DNMT3A knockdown AGS cells (**E**), DNMT3B knockdown AGS cells (**F**) were established. **G** The expression of PCDH10 in gastric cancer cells from the Broad Institute CCLE databases. **Fig. S5. A, B** CCK-8 assay and colony formation was performed to determine the effect of RNF180 knockdown on cell proliferation.

**Additional file 2: Table S1.** Correlations between PCDH10 and clinicopathological features in 40 gastric cancer patients in RNA database. **Table S2.** Correlations between RNF180 and clinicopathological features in 176 gastric cancer patients. **Table S3.** Correlations between RNF180 and clinicopathological features in 176 gastric cancer patients. **Table S4.** The list and sequences of primers used for qRT-PCR. **Table S5.** The list of primary antibodies used.

**Additional file 3.** Supplementary materials and methods.

## Data Availability

The datasets used and/or analyzed during the current study are available from the corresponding author upon reasonable request.

## Abbreviations

CCLE: Cancer Cell Line Encyclopedia
CO-IP: Co-immunoprecipitation
CXCL8: C-X-C motif chemokine ligand 8
DNMT: DNA methyltransferase
EREG: Epiregulin
EWAS: Epigenome-wide association studies
GC: Gastric cancer
GTEx: Genotype-Tissue Expression
iAUC: Integrated area under the ROC curve
IP: Immunoprecipitation
iTRAQ: Isobaric tags for relative and absolute quantitation
MMP: Matrix metalloproteinase
NGS: Next-generation sequencing
OS: Overall survival
PCDH10: Protocadherin 10
qRT-PCR: Quantitative real-time polymerase chain reaction
RASA4: RAS p21 protein activator 4
RNA-seq: RNA sequencing
RNF: RING finger
shRNAs: Short hairpin RNAs
TGFB1: Transforming growth factor beta 1
TSGs: Tumor suppressor genes
