# Supplementary material for: E3 ubiquitin ligase RNF180 prevents excessive PCDH10 methylation to suppress the proliferation and metastasis of gastric cancer cells by promoting ubiquitination of DNMT1
Source: Clin Epigenetics. 2023 May 5;15:77. doi: 10.1186/s13148-023-01492-y (PMC10163782; doi:10.1186/s13148-023-01492-y)
Supplement: Supplementary file 1 — Additional file 1: Fig. S1. A PCDH10 protein levels in GC cell lines and GES-1. B The global methylation level of PCDH10 in gastric cancer cells from the Broad Institute CCLE databases. C The global methylation level of PCDH10 in gastric cancer tissues, adjacent non-tumor tissues from EWAS database. D The global methylation status of PCDH10 in gastric cancer tissues, adjacent non-tumor tissues confirmed by the NGS methylation analysis. E, F Western blot and qPCR confirmed that DNMT1 knockdown increased the protein and mRNA expression of PCDH10. Fig. S2. A PCDH10 overexpression led to an elevated expression of the cleaved form of caspase- 3 and poly (ADP-ribose) 2 polymerase (PARP) in HGC27 and AGS cells treated with staurosporine, which is an apoptosis-inducing reagent. B PCA for the expression profiles to distinguish the two groups (PCDH10 overexpression and control groups). C Volcano plot of altered gene expression patterns in PCDH10 overexpressed HGC27 cells identified from RNA-seq analysis. Fig. S3. A PCA for the expression profiles to distinguish the two groups (RNF180 overexpression and control groups. B Heatmap of 30 dysregulated genes identified from iTRAQ quantitative analysis. C The significant GO terms in the enrichment analysis of 30 dysregulated genes identified from iTRAQ quantitative analysis. D qPCRanalyses were used to detect the mRNA expression of DNMT1 in RNF180 overexpressed HGC27 cells and RNF180 silencing MKN45 cells. E qPCR analyses were used to detect the mRNA expression of PCDH10 in RNF180 overexpressed HGC27 cells. F Mutation in the RING domain of RNF180 did not affect the protein level of DNMT1 and PCDH10. G, H CCK8 and colonyforming assays confirmed that DNMT1 upregulation partially countervailed the inhibitive effect of RNF180 overexpression on the proliferation of HGC27 cells. I RNF180 lacking the RING domain failed to reverse MG132-induced increase in DNMT1 expression in HGC27 cells. Fig. S4. A Stable PCDH10 overexpressed HGC27 and AGS [file 13148_2023_1492_MOESM1_ESM.pdf]

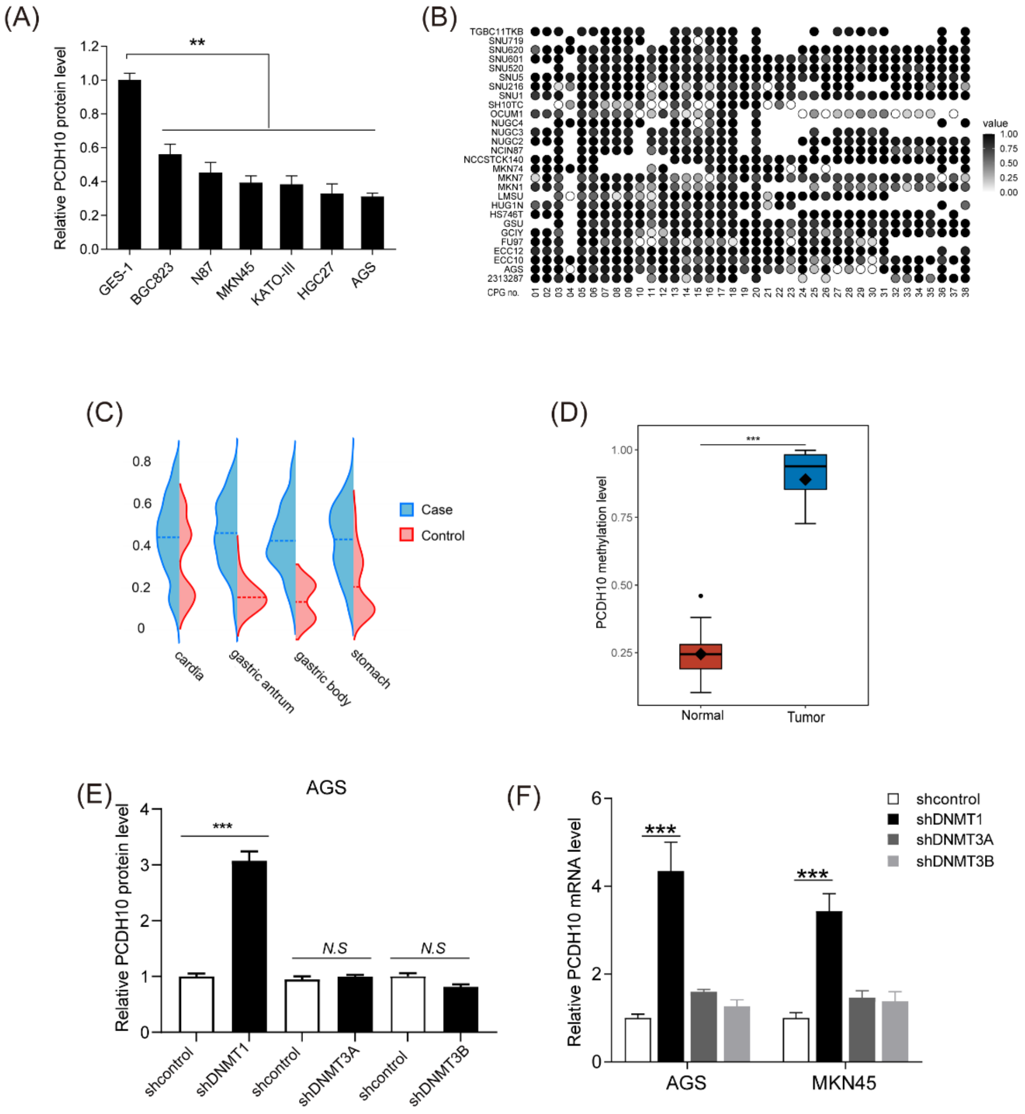

Fig. S1. (A) PCDH10 protein levels in GC cell lines and GES-1. (B) The global methylation level of PCDH10 in gastric cancer cells from the Broad Institute CCLE databases. (C) The global methylation level of PCDH10 in gastric cancer tissues, adjacent non-tumor tissues from EWAS database. (D) The global methylation status of PCDH10 in gastric cancer tissues, adjacent non-tumor tissues confirmed by the NGS methylation analysis. (E, F) Western blot and qPCR confirmed that DNMT1 knockdown increased the protein and mRNA expression of PCDH10.

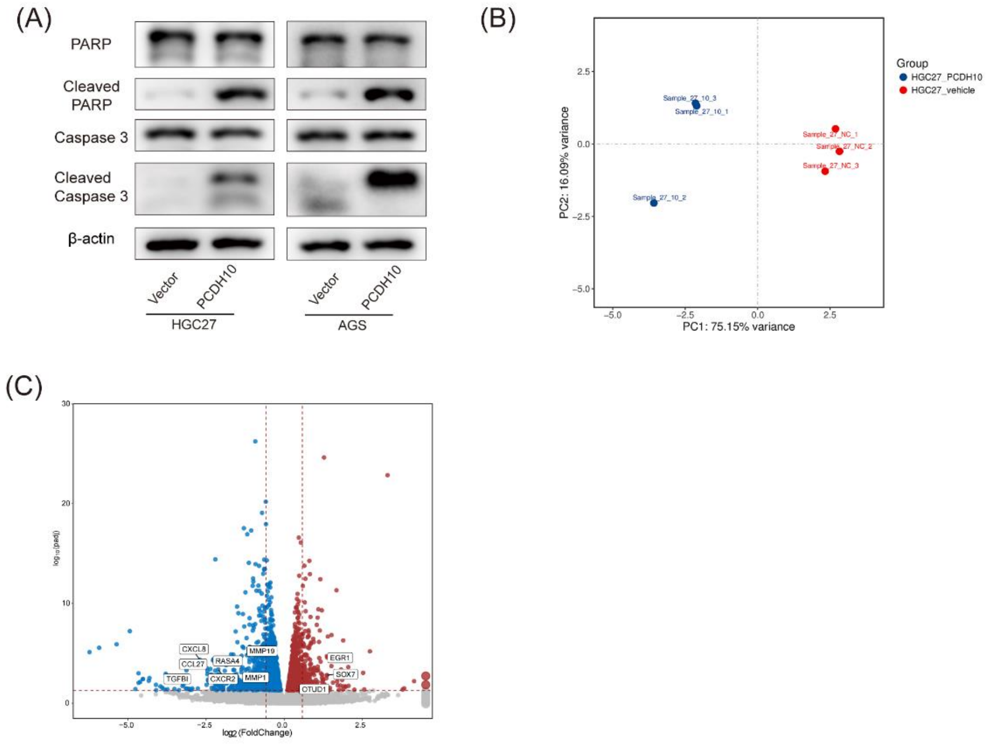

Fig. S2. (A) PCDH10 overexpression led to an elevated expression of the cleaved form of caspase-3 and poly (ADP-ribose) 2 polymerase (PARP) in HGC27 and AGS cells treated with staurosporine, which is an apoptosis-inducing reagent (B) PCA for the expression profiles to distinguish the two groups (PCDH10 overexpression and control groups). (C) Volcano plot of altered gene expression patterns in PCDH10 overexpressed HGC27 cells identified from RNA-seq analysis.

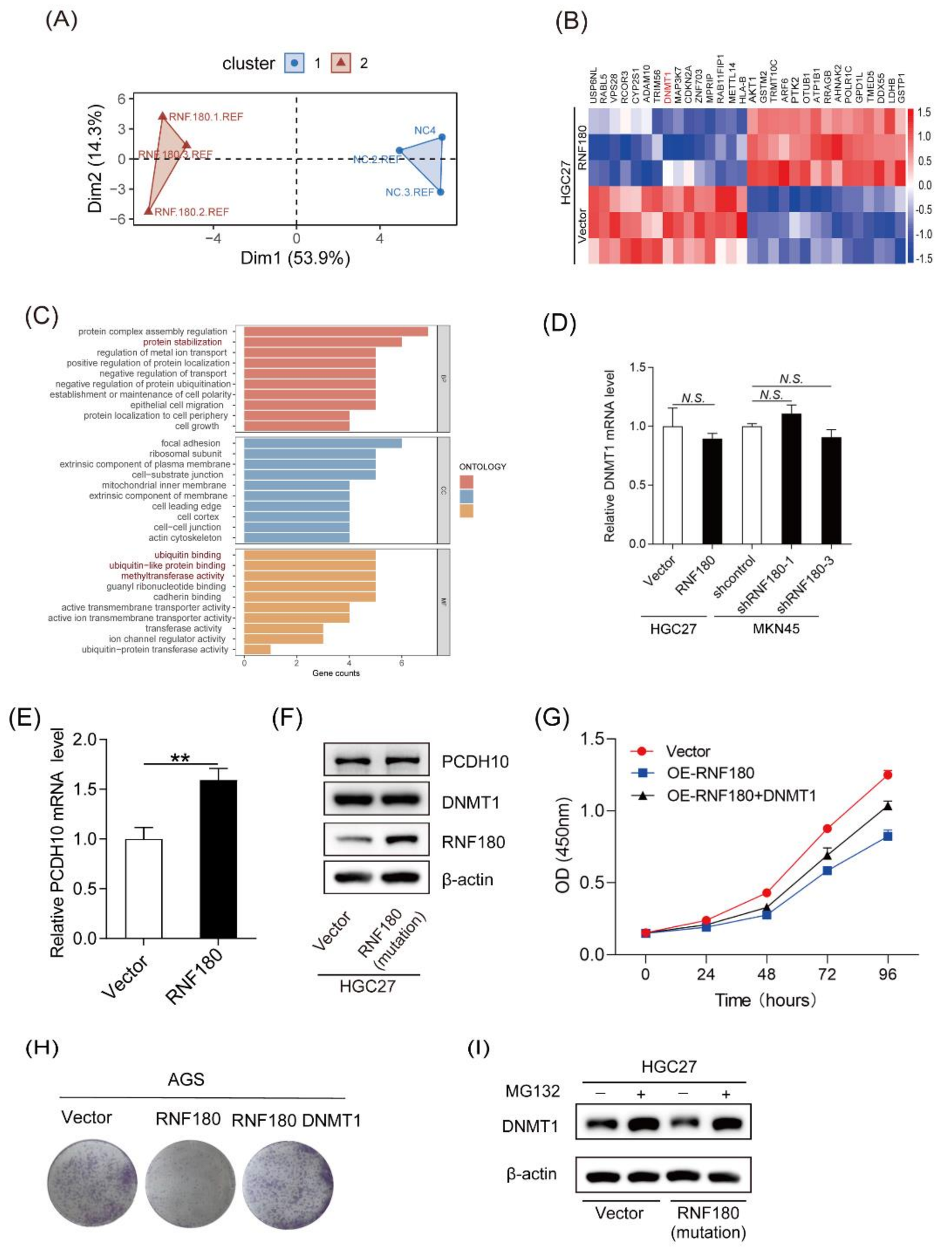

Fig. S3. (A) PCA for the expression profiles to distinguish the two groups (RNF180 overexpression and control groups. (B) Heatmap of 30 dysregulated genes identified from iTRAQ quantitative analysis. (C) The significant GO terms in the enrichment analysis of 30 dysregulated genes identified from iTRAQ quantitative analysis. (D) qPCR analyses were used to detect the mRNA expression of DNMT1 in RNF180 overexpressed HGC27 cells and RNF180 silencing MKN45 cells. (E) qPCR analyses were used to detect the mRNA expression of PCDH10 in RNF180 overexpressed HGC27 cells. (F) Mutation in the RING domain of RNF180 did not affect the protein level of DNMT1 and PCDH10. (G, H) CCK8 and colony-forming assays confirmed that DNMT1 upregulation partially countervailed the inhibitive effect of RNF180 overexpression on the proliferation of HGC27 cells. (I) RNF180 lacking the RING domain failed to reverse MG132-induced increase in DNMT1 expression in HGC27 cells.

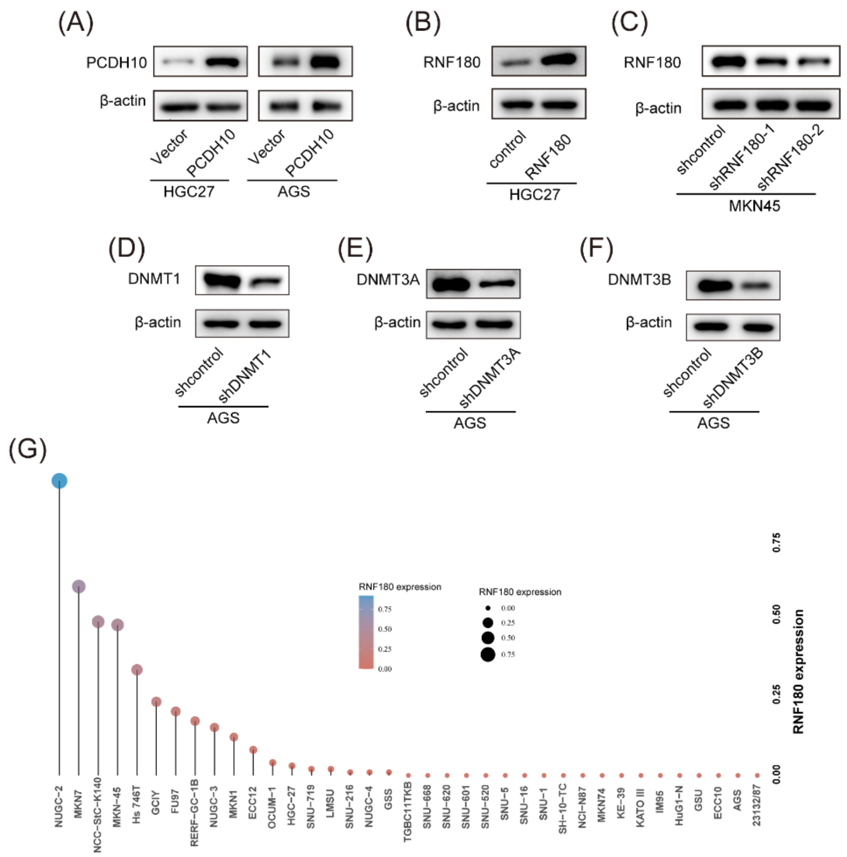

Fig. S4. (A) Stable PCDH10 overexpressed HGC27 and AGS cells were established. The levels of PCDH10 in HGC27 and AGS cells were assessed by western blotting and β-actin was used as a loading control. (B, C) Stable RNF180 overexpressed HGC27 cells (B) and stable RNF180 knockdown MKN45 cells (C) were established. The levels of RNF180 in HGC27 or MKN45 cells were assessed by western blotting and β-actin was used as a loading control. (D, E, F) Stable DNMT1 knockdown AGS cells (D), DNMT3A knockdown AGS cells (E), DNMT3B knockdown AGS cells (F) were established. The levels of DNMT1, DNMT3A and DNMT3B in AGS cells were assessed by western blotting and β-actin was used as a loading control. (G) The expression of PCDH10 in gastric cancer cells from the Broad Institute CCLE databases.

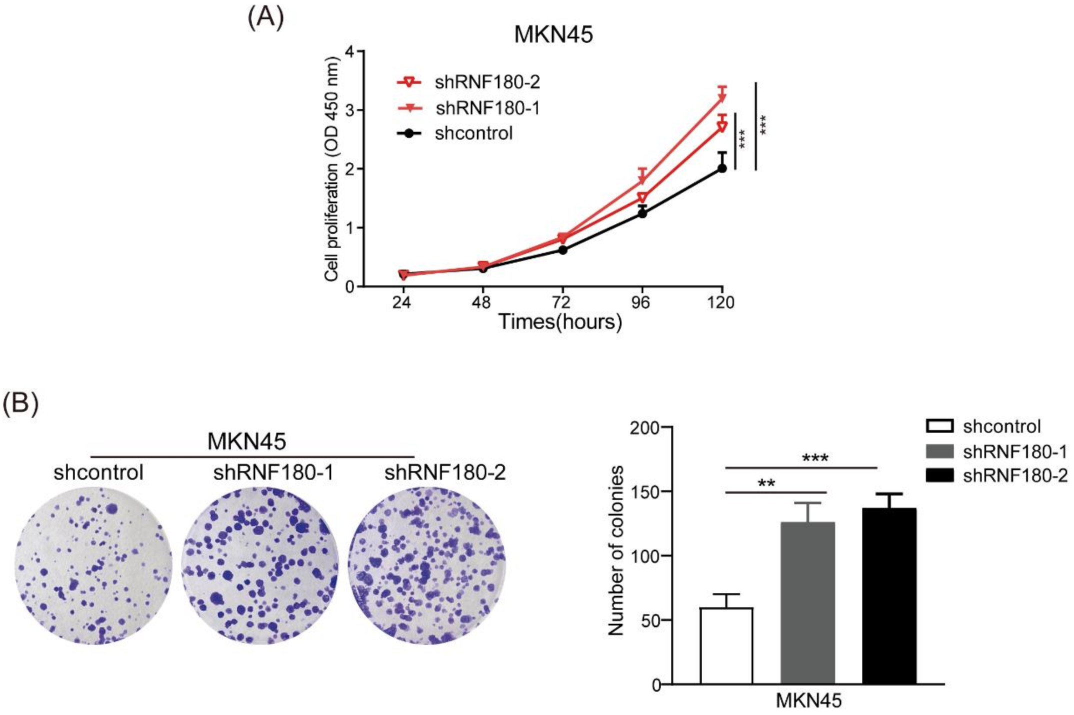

Fig. S5. (A, B) CCK-8 assay and colony formation was performed to determine the effect of RNF180 knockdown on cell proliferation.
