# Supplementary material for: E3 ubiquitin ligase RNF180 prevents excessive PCDH10 methylation to suppress the proliferation and metastasis of gastric cancer cells by promoting ubiquitination of DNMT1
Source: Clin Epigenetics. 2023 May 5;15:77. doi: 10.1186/s13148-023-01492-y (PMC10163782; doi:10.1186/s13148-023-01492-y)
Supplement: Supplementary file 2 — Additional file 2: Table S1. Correlations between PCDH10 and clinicopathological features in 40 gastric cancer patients in RNA database. Table S2. Correlations between RNF180 and clinicopathological features in 176 gastric cancer patients. Table S3. Correlations between RNF180 and clinicopathological features in 176 gastric cancer patients. Table S4. The list and sequences of primers used for qRT-PCR. Table S5. The list of primary antibodies used. [file 13148_2023_1492_MOESM2_ESM.pdf]

Table S1 Correlations between PCDH10 and clinicopathological features in 40 gastric cancer patients in RNA database.

Variable	Number of patients		Chi-square value	p value*
	$PCDH10^{Low}$	$PCDH10^{High}$		
Gender			2.133	0.144
Male	13	17		
Female	7	3		
Age, years			0.114	0.736
≤ 60	7	6		
> 60	13	14		
Tumor size, cm			6.144	0.013
≤ 4	2	9		
> 4	18	11		
Lauren classification			1.616	0.204
intestinal	11	7		
diffuse	9	13		
pT stage			2.626	0.492
T1	0	1		
T2	3	6		
T3	2	2		
T4a	15	11		
T4b	0	0		
pN stage			12.593	0.008
N0	4	4		
N1	1	4		
N2	1	8		
N3a	11	3		
N3b	3	1		
Vascular invasion			0.440	0.507
Yes	8	6		
No	12	14		
Perineural invasion				
Yes	8	7	0.107	0.744
No	12	13		

A chi-square test was used for comparing groups between low and high PCDH10 expression.

*$p < 0.05$ was considered significant.

Table S2 Correlations between RNF180 and clinicopathological features in 176 gastric cancer patients.

Variable	Number of patients		Chi-square value	*p* value*
	RNF180^{Low}	RNF180^{High}		
Gender			0.071	0.789
Male	86	35		
Female	38	17		
Age, years			0.053	0.818
≤ 60	74	32		
> 60	50	20		
Tumor size, cm			5.184	0.023
≤ 4	53	32		
> 4	71	20		
Lauren classification			0.304	0.582
intestinal	54	25		
diffuse	70	27		
Type of gastrectomy			0.369	0.831
Distal subtotal	61	23		
Proximal subtotal	19	9		
Total	44	20		
pT stage			2.674	0.475
T1	0	0		
T2	12	4		
T3	5	2		
T4a	101	40		
T4b	6	6		
pN stage			11.145	0.025
N0	18	13		
N1	11	11		
N2	31	13		
N3a	43	9		
N3b	21	6		

A chi-square test was used for comparing groups between low and high RNF180 expression.

*$p < 0.05$ was considered significant.

Table S3 Correlations between DNMT1 and clinicopathological features in 176 gastric cancer patients.

Variable	Number of patients		Chi-square value	*p* value*
	DNMT1^{Low}	DNMT1^{High}		
Gender			0.246	0.620
Male	51	70		
Female	21	34		
Age, years			0.040	0.876
≤ 60	44	62		
> 60	28	42		
Tumor size, cm			4.917	0.027
≤ 4	42	43		
> 4	30	61		
Lauren classification			2.083	0.149
intestinal	37	42		
diffuse	35	64		
Type of gastrectomy			2.370	0.306
Distal subtotal	31	53		
Proximal subtotal	10	18		
Total	31	33		
pT stage			8.430	0.032
T1	0	0		
T2	12	4		
T3	3	4		
T4a	53	88		
T4b	4	8		
pN stage			5.356	0.235
N0	16	15		
N1	10	12		
N2	21	23		
N3a	16	36		
N3b	9	18		

A chi-square test was used for comparing groups between low and high RNF180 expression.

*$p < 0.05$ was considered significant.

Table S4 The list and sequences of primers used for qRT-PCR.

PCDH10	5'-TCTCCAACGGAAGCATTTTGTCC/CTATGTCGGCTTCCTGGAATGC-3'
RNF180	5'-CCTTCCAGAATGGATAAGCTGCC/GCCACAGGAACATTTTGGAGTGC-3'
DNMT1	5'-AGGTGGAGAGTTATGACGAGGC/GGTAGAATGCCTGATGGTCTGC-3'
MMP-1	5'-ATGAAGCAGCCCAGATGTGGAG/TGGTCCACATCTGCTCTTGGCA-3'
MMP-19	5'-GCAGTAGTGAACTGGATGCCATG/CAAAGGGCAGACACTCGGAACA-3'
EREG	5'-CTTATCACAGTCGTCGGTTCCAC/GCCATTCAGACTTGCGGCAACT -3'
RASA4	5'-GCTGAAGGACTTCATCACCAAGC/TTGCCCTTGGTCCTGTGGATGA-3'
CXCL8	5'-GAGAGTGATTGAGAGTGGACCAC/CACAACCCTCTGCACCCAGTTT-3'
TGFB1	5'-TACCTGAACCCGTGTTGCTCTC/GTTGCTGAGGTATCGCCAGGAA-3'
GAPDH	5'-GTCTCCTCTGACTTCAACAGCG/ACCACCCTGTTGCTGTAGCCAA-3'

Table S5 The list of primary antibodies used.

Target	Usage	Source	Catalog number	Dilution
RNF180	WB	Genetex	GTX119301	1:1000
RNF180	IF	NOVUS	H00285671-M05	1:200
PCDH10	WB	NOVUS	H00057575-M01	1:1000
PCDH10	IHC	Genetex	GTX117862	1:300
DNMT1	WB	CST	5032S	1:1000
DNMT1	IHC/IF	Genetex	GTX116011	1:300
DNMT3A	WB	CST	32578S	1:1000
DNMT3B	WB	CST	57868T	1:1000
FLAG	WB	CST	14793S	1:1000
Ubiquitin	WB	Abcam	Ab134953	1:1000
β -actin	WB	CST	8457S	1:1000
