# Supplementary material for: E3 ubiquitin ligase RNF180 prevents excessive PCDH10 methylation to suppress the proliferation and metastasis of gastric cancer cells by promoting ubiquitination of DNMT1
Source: Clin Epigenetics. 2023 May 5;15:77. doi: 10.1186/s13148-023-01492-y (PMC10163782; doi:10.1186/s13148-023-01492-y)
Supplement: Supplementary file 3 — Additional file 3. Supplementary materials and methods. [file 13148_2023_1492_MOESM3_ESM.pdf]

Additional file 3. Supplementary materials and methods

Patient selection

The inclusion criteria were as follows: 1) pathologic diagnosis of primary advanced gastric adenocarcinoma (pT2-4); 2) no esophagogastric junction cancer; 3) no distant metastasis; 4) pathologically negative resection margins (R0 resection); 5) no residual GC after surgery; 6) no neoadjuvant chemotherapy or radiotherapy; 7) no other synchronous malignancy or previous history of gastrectomy.

Data availability

We aimed to identify PCDH10 with critical pathogenetic roles in GC, by data mining of the TCGA and Genotype-Tissue Expression (GTEx) databases with the Assistant for Clinical Bioinformatics (https://www.aclbi.com/static/index.html#/). The Epigenome-wide Association Studies (EWAS) database (https://bigd.big.ac.cn/ewas/index), Shiny Methylation Analysis Resource Tool (SMART) App (http://www.bioinfo-zs.com/smartapp) and Broad Institute Cancer Cell Line Encyclopedia (CCLE) database (https://portals.broadinstitute.org/ccle) were used to explore the methylation status of PCDH10 and the relationship between methylation status and mRNA expression of PCDH10.

Cell culture

The human GC cell lines MKN-45, HGC-27, BGC-823, and KATO III, and normal human gastric epithelial cell line GES-1 were obtained from Cancer Research Institute of Beijing

University. Another GC cell lines AGS and N87 were purchased from the Type Culture Collection of the Chinese Academy of Sciences (Shanghai, China). All GC and GES-1 cell lines, excluding AGS cell lines were cultured in RPMI 1640 medium (Gibco, USA) supplemented with 10% fetal bovine serum (OriCell, New Zealand) and 1% penicillin-streptomycin (Gibco, USA). AGS was cultured in F12 Nutrient Mixture medium (Gibco, USA) supplemented with 10% fetal bovine serum and 1% penicillin-streptomycin. All cells were cultured at 37 °C in a thermostatic incubator containing 5% CO_2. All cell lines were authenticated by STR profiling within three years, and the experiments were conducted using mycoplasma-free cells.

qRT-PCR

According to the manufacturer's instructions, total RNA was extracted from tissues and cultured cells with TRIzol reagent (Invitrogen, USA) and reverse-transcribed into cDNA using a PrimeScript RT Reagent Kit (Takara, Japan). The cycling conditions were 94 °C for 5 min, 40 cycles of 95◦C for 5 s, 60◦C for 20 s, and 95◦C for 15 s, 60◦C for 1min, 95◦C for 30 s, 60◦C for 15 s. Triplicates were performed for each reaction. The primer sequences for qRT-PCR are shown in Additional file 1: Table S4.

Western blotting

Whole-cell extracts were prepared by lysing cells in RIPA buffer (Millipore, USA) supplemented with phosphatase inhibitor and protease inhibitor (Millipore, USA). Western

blots and sodium dodecyl sulfate polyacrylamide gel electrophoresis (SDS-PAGE) were used to separate protein lysates and detect target proteins. The primary antibodies used are listed in Additional file 1: Table S5.

Gene chip detection analysis

Stable HGC27-RNF180 cells and HGC27-vector cells were were lysed in Trizol reagent (Invitrogen) and total RNA was extracted according to instructions. The global mRNA expression profiles were detected using a GeneChip® PrimeView™ Human Gene Expression Array (Affymetrix, Santa Clara, CA, USA). The raw data were analyzed using GeneSpring software version 12.5 (Agilent Technologies, Santa Clara, CA, USA) and normalized using a quantile algorithm. Gene expression changes ≥ 2.0-fold higher or lower and $P < 0.05$ were considered as biologically significant. Differences in mRNA expression were verified by qRT-PCR.

RNA sequencing (RNA-seq) and analysis

Three replicates for stable HGC27-PCDH10 and HGC27-vector cells were seeded and collected and total RNA was extracted using the RNeasy kit (Qiagen, Germany) according to the manufacturer's instructions. RNA integrity was examined using an Agilent 2100 Bioanalyzer (Agilent Technologies, Santa Clara, CA, USA). Following the sample integrity check, the subsequent analysis was conducted only for samples with integrity ≥ 7. Library construction and transcriptome sequencing and analysis were performed by OE Biotech Co., Ltd. (Shanghai, China). Thresholds for significantly differential expression were set as

|log2FoldChange|<1 and $P < 0.05$.

Next-generation sequencing (NGS) approach for methylation analysis

Total DNA was extracted using a QIAamp® DNA Mini Kit (Qiagen, Germany) according to the manufacturer's protocol. Raw reads were filtered according to two steps: 1) Removing adaptor sequence if reads contains by cutadapt (v 1.2.1); 2) Removing low quality bases from reads 3' to 5' (Q < 20) by PRINSEQ-lite（v 0.20.3); 3）Bismark (version v0.22.1)(www.bioinformatics.babraham.ac.uk/projects/)was used for CpG detect with defualt parameters. Quantitative DNA methylation analysis of PCDH10 was then performed using NGS with a MiSeq System (Illumina, San Diego, CA, USA), using reversible-terminator sequencing coupled with synthesis technology to create massively parallel sequencing environments.

iTRAQ based proteomics

RNF180 expression and empty vectors were transfected into HGC-27 cells. Samples were prepared for LC-MS/MS iTRAQ analysis as reported previously.[1] ITRAQ analysis was performed using pooled samples digested according to the FASP protocol and labeled using an 8-plex iTRAQ reagent (Applied Biosystems). Protein identification and iTRAQ were conducted using Mascot version 2.2 (Matrix Science, USA) and Proteome Discoverer software version 1.4 (Thermo Scientific, USA) based on the UniProt Human Database. The final proteins considered differentially expressed were filtered in accordance with a P-value < 0.05 and fold change > 1.5 or < 0.67 compared to the control group.

Immunoprecipitation (IP) and co-immunoprecipitation (Co-IP)

IP and co-IP were performed using a Pierce Co-IP Kit (Thermo Fisher Scientific) as per the manufacturer's instructions, with the following antibodies: anti-Flag (Sigma, Saint Louis, MO, USA), anti-DNMT1 (GTX116011; Genetex), anti-RNF180 (GTX119301; GeneTex), mouse immunoglobulin G (Sigma), and anti-ubiquitin (Ab134953; Abcam).

Cell Counting Kit-8 (CCK-8) and colony-formation assays

Briefly, for cell proliferation, cells were inoculated into 96-well plates (1000 cells per well) and 10 μl of CCK-8 reagent was added to the wells after 1, 2, 3, and 4 days. The plates were incubated for 2 h, and the absorbance was determined at 490 nm. For colony-formation assays cells were seeded in a 6-cm culture dish (750 cells) and the culture medium was refreshed every 3 days for 2 weeks. The cells were then washed with phosphate-buffered saline (PBS), fixed with 4% paraformaldehyde, and stained with 0.4% crystal violet for 15 min. The number of colonies containing >10 cells was counted manually and averaged over duplicate wells.

Cell cycle and apoptosis

Stable gastric cancer cells were collected, washed with phosphate-buffered saline twice, and stained with propidium iodide (Sigma–Aldrich) at a final concentration of 17.4 lg/mL. Cells were analyzed using fluorescenceactivated cell sorting analysis (FACS Calibur; BD Biosciences, San Jose, CA), and data were analyzed using ModFit LT software (Verity Software House, Topsham, ME). The apoptosis analysis was determined by PE Annexin V kit (PharMingen, BD Biosciences). Cells were stained with PE Annexin V and 7-AAD, and data

were analyzed using EXPO32 ADC Cellquest Analysis Software (Beckman Coulter, Brea, CA).

Cell migration and invasion assay

Cell migration and invasion was assayed using Transwell chambers (6.5 mm; Corning, NY, USA) with 8-μm pore membranes; for cell invasion assays, the upper face of the membrane was covered with 70 μl of Matrigel (1 mg/ml) (BD Biosciences, NJ, USA). The lower chamber was filled with 1500 μl of lower medium (medium with 20% FBS). The cells (5×10^4 cells/well) were suspended in 200 μl of upper medium (medium without FBS) and were plated into the upper chamber. After 24 and 36 hours, the number of crystal violet-stained cells on the undersurface of the polycarbonate membranes was visually counted in five random fields at 100 × magnification.

Protein half-life detection

HGC27 cells were infected with the lentiviral vector encoding RNF180 (LV-RNF180) or with negative control vector, and MKN45 cells were infected with lentiviral vectors carrying RNF180-shRNA or empty vector, as described above. A total of 10 mg/mL cycloheximide (HY-12320, MCE) was added to the culture medium. Following exposure to cycloheximide, cells were lysed in RIPA buffer supplemented with protease and phosphatase inhibitors at the indicated time points. Furthermore, cells were also incubated with 10 μM MG132 (HY-13259, MCE) for an additional 10 h and then collected for western blotting to determine the amount of DNMT protein.

Immunofluorescence

Immunofluorescence was used to detect the location and expression of target proteins, as described previously.[2] Briefly, cells were seeded onto glass coverslips in 24-well plates, washed with PBS. After being fixed with 4% paraformaldehyde, incubated in 0.3% Triton X-100 and blocked with 5% FBS, cells were incubated with primary antibodies at 4 °C overnight, followed by incubation with the appropriate secondary antibody (Yeasen, Shanghai, China) for 1 h. The nuclei were counterstained with 4, 6-diamidino-2-phenylindole (DAPI, Yeasen, Shanghai, China). Immunofluorescence was performed using rabbit anti-DNMT1 (1:100) (5032S; CST) and mouse anti-RNF180 (1:100) (H00285671-M05; Novus).

Immunohistochemical (IHC) staining

Tissue staining was performed as previously published.[3] Immunohistochemistry was performed using rabbit anti-PCDH10 (1:200) (GTX117862; GeneTex), anti-DNMT1 (1:200) (GTX116011; GeneTex), and rabbit anti-RNF180 antibody (1:150) (GTX119301; GeneTex). PCDH10, DNMT1, and RNF180 expression were assessed according to the average intensity of positive cells (−, none; +, weak; ++, intermediate; +++, strong). Intensity scores of ++ and +++ were considered to represent high staining.

Animal experiments

Xenograft experiments in nude mice were approved by the Animal Experimentation Ethics Committee of Tianjin Medical University Cancer Institute and Hospital. GC cells stably

expressing PCDH10 and control cells were harvested and suspended in PBS. A total of twelve female BALB/c mice between 4 and 6 weeks were randomly divided into two groups, each with six mice, and subcutaneous injections of 4×106 cells were administered to each mouse in the inguinocrural region. Measurements of tumor dimensions were taken manually three times a week, and tumor volumes calculated using the formula (width2 x length/2). Approximately 4 weeks after injection, the mice were sacrificed, and the tumors were dissected and weighed. For *in vivo* tumor metastasis studies, 4×10^6 luciferase-tagged GC cells infected with LV-PCDH10, or the corresponding negative control were suspended in 200 μl of PBS and injected into the tail vein of nude mice. With the IVIS Spectrum in vivo imaging system, the bioluminescence intensity was measured after 8 weeks of injection. For the assessment of the number of metastases, the lung tissues were fixed in 4% PFA and stained in hematoxylin and eosin (H&E).

REFERENCE

1. Buchel C, Miedl S, Sprenger C. Hedonic processing in humans is mediated by an opioidergic mechanism in a mesocorticolimbic system. *Elife* 2018;7.

2. Sáenz JB, Vargas N, Mills JC. Tropism for Spasmolytic Polypeptide-Expressing Metaplasia Allows Helicobacter pylori to Expand Its Intragastric Niche. *Gastroenterology* 2019;156: 160-74.e7.

3. Deng J, Liang H, Zhang R, Hou Y, Liu Y, Ying G, Pan Y, Hao X. Clinical and experimental role of ring finger protein 180 on lymph node metastasis and survival in gastric cancer. *Br J Surg* 2016;103: 407-16.
